# Characterization of CRISPR Spacer and Protospacer Sequences in *Paenibacillus larvae* and Its Bacteriophages

**DOI:** 10.3390/v13030459

**Published:** 2021-03-11

**Authors:** Casey Stamereilers, Simon Wong, Philippos K. Tsourkas

**Affiliations:** School of Life Sciences, University of Nevada, Las Vegas, NV 89154, USA; casey.stamereilers@unlv.edu (C.S.); wongs3@unlv.nevada.edu (S.W.)

**Keywords:** American foulbrood, *Paenibacillus larvae*, CRISPR, spacers, protospacers, phages, bacterial immunity

## Abstract

The bacterium *Paenibacillus larvae* is the causative agent of American foulbrood, the most devastating bacterial disease of honeybees. Because *P. larvae* is antibiotic resistant, phages that infect it are currently used as alternative treatments. However, the acquisition by *P. larvae* of CRISPR spacer sequences from the phages could be an obstacle to treatment efforts. We searched nine complete genomes of *P. larvae* strains and identified 714 CRISPR spacer sequences, of which 384 are unique. Of the four epidemiologically important *P. larvae* strains, three of these have fewer than 20 spacers, while one strain has over 150 spacers. Of the 384 unique spacers, 18 are found as protospacers in the genomes of 49 currently sequenced *P. larvae* phages. One *P. larvae* strain does not have any protospacers found in phages, while another has eight. Protospacer distribution in the phages is uneven, with two phages having up to four protospacers, while a third of phages have none. Some phages lack protospacers found in closely related phages due to point mutations, indicating a possible escape mechanism. This study serve a point of reference for future studies on the CRISPR-Cas system in *P. larvae* as well as for comparative studies of other phage–host systems.

## 1. Introduction

American foulbrood (AFB) is the most destructive bacterial disease in honeybees (*Apis mellifera)* [1]. It is caused by the Gram-positive, spore-forming bacterium *Paenibacillus larvae* and afflicts honeybee larvae. AFB outbreaks usually occur when larvae ingest food contaminated with *P. larvae* spores; as few as ten spores are enough to trigger a fatal infection [1,2]. The *P. larvae* spores germinate and rapidly proliferate in the larval midgut, lysing the infected larva from the inside within 12 h of ingestion [1,3]. As worker bees remove the deceased larvae, they inadvertently spread millions of spores through the hive [1,4], with the result being complete hive collapse on the order of 7–12 days [1,5]. *P. larvae* is classified into five genotypes based on enterobacterial repetitive intergenic consensus (ERIC) primers; the genotypes correlate with phenotypic differences [6]. The ERIC I and ERIC II genotypes are found worldwide and cause virtually all AFB outbreaks [1,6], with ERIC I accounting for the majority [6]. The ERIC III and ERIC IV strains are closely related genetically, but have not been isolated in the field for decades, while ERIC V was isolated from a field honey sample in 2020 [6]. Traditionally, AFB has been treated using antibiotics (tylosin, lincomycin, and oxytetracycline), but antibiotic resistant strains of *P. larvae* are now widespread [1,6,7,8,9]. Additionally, antibiotic residues are found in honey after antibiotic treatments [7], and thus several countries have banned the use of antibiotics to treat AFB [5]. *P. larvae* spores are extremely durable, being resistant to heat and cold, and can remain infectious for decades [1,10]. If an infection is not treatable with antibiotics, total incineration of the hive and any beekeeping equipment is required [1].

The problems associated with antibiotic treatment of AFB have led to interest in phage therapy as an alternative treatment. Three studies have shown that phages are effective at lysing *P. larvae* in laboratory settings [11,12,13]; in one of these studies, a lysis screen that tested the ability of 29 phages to lyse 11 *P. larvae* strains showed that there is considerable variability in lytic ability and host range between phages [13]. Additionally, one field study has successfully used phages to treat AFB in the field [14]. The first *P. larvae* phage genome was sequenced in 2013 [15], and the current number of sequenced phage genomes currently stands at 49 [11,15,16,17,18,19,20,21,22]. Sequencing the genomes of *P. larvae* phage is important so as to identify *P. larvae* phage proteins and their function, uncover the mechanisms by which the phages lyse *P. larvae* or enter lysogeny, and identify potentially dangerous or novel phage proteins. For example, a putative toxin found in four phage genomes has been identified as contributing to the pathogenicity of the ERIC I strain [23]. Two comparative genomic studies have classified sequenced *P. larvae* phages into clusters, assigned putative functions to phage proteins, and identified conserved genes [24,25]. Genome length ranges from 35 kbp to 55 kbp, and the 49 phages are grouped into four clusters and two singletons based on average nucleotide sequence identity (ANI) [22,25]. All sequenced *P. larvae* phages are temperate [22,25]. Roughly half of *P. larvae* phage proteins have putative function [25]. All sequenced *P. larvae* phages encode a N-acetylmuramoyl-L-alanine amidase that they use to pierce their host’s peptidoglycan cell wall [22,25]. Three studies have focused on the N-acetylmuramoyl-L-alanine amidase [26,27,28]; in one of these studies the amidase was successfully used to rescue honeybee larvae infected with *P. larvae* [27]. The reader is referred to ref. 29 for a review of *P. larvae* phages [29].

Despite the increase in information on *P. larvae* and the phages that infect them, no study has yet investigated the existence or distribution of clustered regularly interspaced short palindromic repeats (CRISPR) spacer sequences in *P. larvae* and *P. larvae* phages. CRISPR is a bacterial and archaeal adaptive immune system that neutralizes invading phages and plasmids by cutting foreign DNA at specific locations [30,31,32]. These specific locations, called protospacers, are acquired from phage genomes during an infection and introduced into the bacterial CRISPR locus as CRISPR spacer sequence [33]. Protospacers are acquired from a region of the phage genome that is flanked by a protospacer adjacent motif (PAM), a 2–5 base pair sequence, which varies in its sequence across bacteria and archaea [34,35,36,37]. The PAM sequence differs from the palindromic repeat sequence in the CRISPR locus of the host genome, eliminating the risk of self-targeting or self-cleaving of the hosts’s genome [38]. In subsequent phage infections, the host can use its previously acquired spacers as targets for complementary binding to the phage genomes; if such binding occurs, the phage DNA is cleaved, thereby neutralizing the infection [39,40,41,42,43,44]. The presence of phage spacer sequences in the *P. larvae* genome could thus compromise the efficacy of phages as treatment agents.

In this study, we identified CRISPR spacer sequences in *P. larvae* genomes, searched *P. larvae* phage genomes for spacer sequences, and assessed the distribution of CRISPR spacer sequences in *P. larvae* strains and *P. larvae* phages. This study serves as a point of reference for future experimental studies on the relationship between the presence of spacers and phage lytic ability, as well as for comparative studies of spacer distribution in other host–phage systems.

## 2. Materials and Methods

The complete genome sequences of nine *P. larvae* strains and 49 *P. larvae* phages were obtained through a search of NCBI GenBank for complete genome sequences. CRISPR spacer sequences were identified using the program CRISPRfinder (https://crispr.i2bc.paris-saclay.fr/Server/ accessed on 12 February 2021) [45], with default settings. CRISPRfinder outputs spacer information as “confirmed” or “questionable”; only “confirmed” spacers were included in the analysis. “Questionable” spacers were also investigated but none were found in the phage genomes. Prophages in the *P. larvae* strains were identified PHASTER, with default settings [46,47].

To search the phage genomes for the spacer sequences, we developed a Python script that searched the phage genomes for all spacer sequences identified with CRISPRfinder. A file containing the 49 sequenced phage genomes and a file containing the spacers were compiled. Each spacer was searched for in each of the phage genomes. A match was made if the spacer sequence was found in the phage genome sequence. The approach used here was limited to exact string matches. The Python script then removed instance of spacers found in more than one strain to establish the list of unique spacers. PAM sequences were searched for by generating multiple alignments of the 10 bases upstream and downstream of the spacer sequences and the PAM sequence was identified using WebLogo [48].

## 3. Results

### 3.1. Distribution of CRISPR Spacer Sequences in P. larvae Strains

The *P. larvae* strains used in this analysis, along with their NCBI accession numbers, are listed in Table 1. Searching these *P. larvae* genomes with CRISPRfinder revealed 714 spacer sequences across all nine sequenced *P. larvae* strains (Table 1). The full list of spacers found in the nine sequenced *P. larvae* strains is given in Supplementary Table S1. Some strains have duplicate spacers; thus the number of unique spacers in each strain is slightly smaller. The distribution of spacers is highly uneven (Table 1), with strains SAG 10367 (ERIC II) and DSM 106052 (ERIC V) having more than 100 spacers, and strains ATCC 9545 (ERIC I), DSM 7030 (ERIC I), and DSM 25430 (ERIC II), fewer than 20. In general, the epidemiologically important ERIC I and ERIC II strains (with the exception of SAG 10367) have noticeably fewer spacers than the ERIC III-V strains. Though the ERIC I strains ATCC 9545 and DSM 7030 have the same number of CRISPR arrays and spacers, they do not share spacers. The number of CRISPR arrays ranges from one to seven, with most strains having six to seven arrays, ranging in size from three to 30 spacers. Some spacers are present in multiple *P. larvae* strains; their distribution is shown in Table 2. Approximately two-thirds of spacers are found in only one *P. larvae* strain, while two spacers are found in eight of nine *P. larvae* strains. The ERIC III strain LMG 16252, and the ERIC IV strains ATCC 13537, CCM 38, and LMG 16247 are all closely related and generally share the same spacers, which accounts for the 83 spacers found in four strains in Table 2.

The number of intact, incomplete, and questionable prophages in each *P. larvae* strain, and the number of spacers in intact, incomplete, and questionable prophages is shown in Table 3. No spacers were found in intact prophages; only three spacers were found in incomplete prophages; and four in questionable prophages. Considering the number of spacers and the number of prophages in *P. larvae* strains, the number of spacers in prophage regions is extremely low.

### 3.2. CRISPR Spacer Sequence Identification in P. larvae Phage Genomes

**Figure 1:**
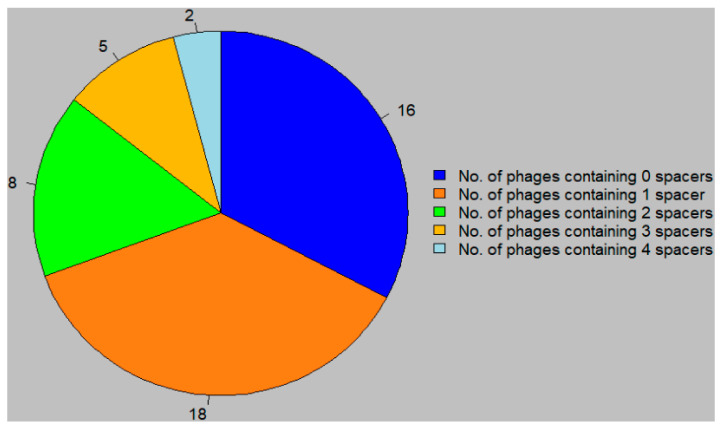
Distribution of *P. larvae* phages by number of unique protospacers.

When spacers occurring in multiple strains are accounted for, there are 384 unique spacers across all nine *P. larvae* strains. The 384 spacers were searched for in the 49 sequenced *P. larvae* phage genomes using a Python script, resulting in the identification of 57 spacer sequences (i.e., protospacers) in the 49 phage genomes. The distribution of the phages by number of protospacers is shown in Figure 1. About a third of sequenced *P. larvae* phages do not contain any protospacers, about a third contain one protospacer, and about a third contain more than one protospacer; two *P. larvae* phages contain a maximum of four protospacer sequences.

**Figure 2:**
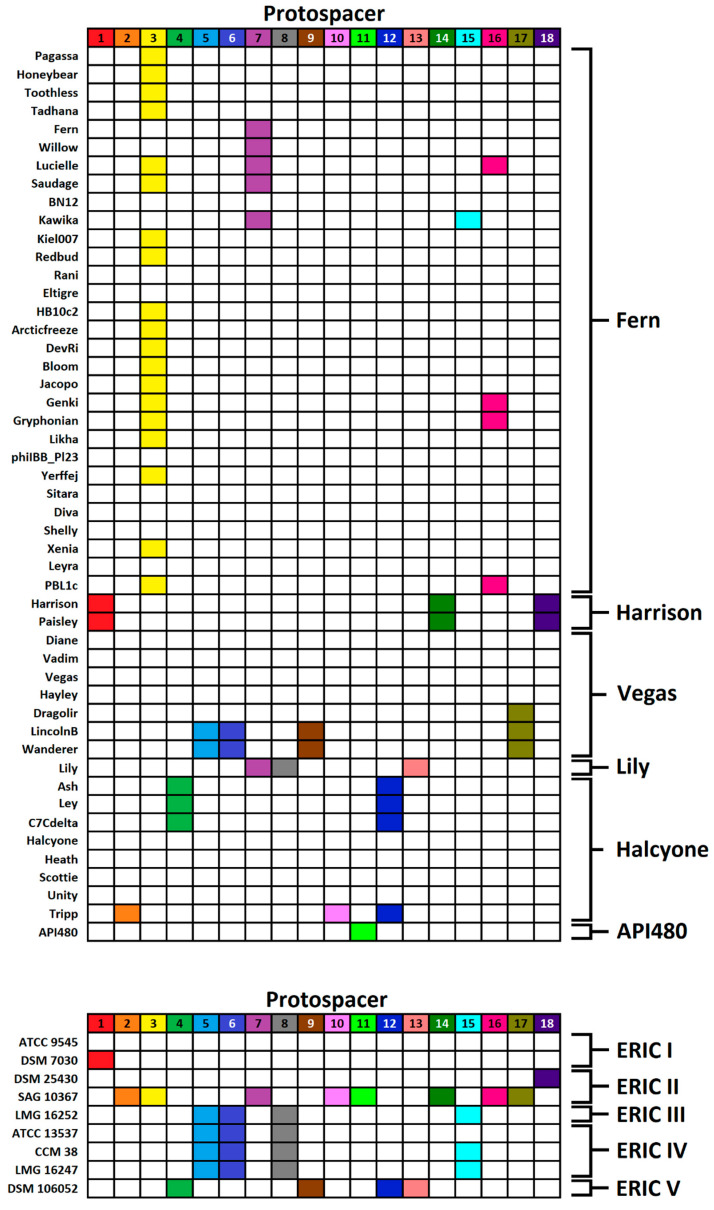
Distribution of the 18 protospacer sequences in *P. larvae* phages and *P. larvae* strains.

After accounting for protospacer sequences found in more than one phage, a total of 18 unique protospacer sequences were identified in the phage genomes. The distribution of these protospacers in *P. larvae* strains and *P. larvae* phages is shown in Figure 2. Phage clusters and ERIC genotypes are shown in brackets on the right. The phages are grouped by genomic clusters, based on whole-genome average nucleotide sequence identity (ANI). 

The current classification of sequenced *P. larvae* phages consists of four clusters and two singletons [22,25]. Clusters are named after a representative phage from each cluster. The largest cluster is the Fern cluster (30 members), followed by the Halcyone cluster (eight members), the Vegas (seven members), the Harrison cluster (two members), and two singletons, Lily and API480. The distribution of protospacers juxtaposed with the 49*49 ANI matrix is included as Supplemental Figure S1. 

As seen in Figure 2, with the exception of SAG 10367, the ERIC I and ERIC II strains have noticeably fewer protospacers compared to the ERIC III–V strains (just as they have fewer spacers in general, per Table 1). Strain ATCC 9545 does not contain any protospacers, while DSM 7030 and DSM 25430 each contain only one protospacer, in both cases in phages Harrison and Paisley. Strain SAG 10367 contains a total of eight unique protospacers from 30 phages that collectively span all four phage clusters and the singletons Lily and API 480. SAG 10367 thus not only contains the most spacers and protospacers, but its protospacers are also the most diverse in terms of the phages they are recruited from. The 18 unique protospacers are generally unique to a *P. larvae* strain, the sole exception being protospacers 5, 6, 8, and 15, which are shared between the closely related ERIC III and ERIV IV strains LMG 16252 ATCC 13537 CCM 38 and LMG 16247 (ATCC 13537 is missing protospacer 15). Strain DSM 106052 (ERIC V, isolated in 2020) contains four unique protospacers not found in other strains, and identified in three different phage clusters.

All phage clusters/singletons have a phage containing at least one of the 18 protospacers, however the phages of the Vegas subcluster (phages Diane, Vadim, Vegas, Hayley) and the Halcyone subcluster (phages Halcyone, Heath, Scottie, Unity) are lacking any protospacers. Phages LincolnB and Wanderer have the most protospacers (four), followed by Harrison, Paisley, Lucielle, Lily, and Tripp, each with three protospacers. Of the phages that contain multiple protospacers, these are roughly evenly split between seven phages whose protospacers are found in multiple strains (phages LincolnB, Wanderer, Tripp, Lily, Harrison, Paisley, Kawika), and eight phages (phages Lucielle, Saudage, Genki, Gryphonian, PBL1c, Ash, Ley, C7 Cdelta), whose protospacers are exclusively found in strain SAG 10367.

All but one of the 18 unique protospacer sequences are unique to one of the six phage clusters/singletons, the sole exception being the SAG 10367 protospacer 7, which is found in the Fern cluster and the singleton Lily. Protospacer 3, also from SAG 10367, is the most widely distributed, found in 19 phages. Six protospacers (2, 8, 10, 11, 13, and 15) are unique to one phage. Protopacers 1, 14, and 18 are unique to the very closely related phages Harrison and Paisley (98% ANI); protospacers 5, 6, and 9 are unique to the very closely related phages LincolnB and Wanderer (99% ANI); and protospacers 4 and 12 are unique to the closely related phages Ash, Ley, and C7 Cdelta (all with <96% ANI to each other). Thus, 14 of the 18 protospacers are unique to either one phage or small groups of very closely related phages. 

Protospacer 3 is of particular interest due to its high frequency in the Fern cluster and its location in the large terminase gene, which is highly conserved among cluster members [25]. In spite of this, 11 of the 30 phages in the Fern cluster lack this protospacer. This is the case with even very closely related phages: Phages Kiel007 and Redbud both contain protospacer 3, but Rani does not, despite having >99% ANI with Kiel007 and Redbud (Supplemental Figure S1). Similarly, phage Xenia contains protospacer 3 while phage Shelly does not, despite 99.5% ANI (Supplemental Figure S1). This is also the case with protospacer 16, which is found in the conserved tail tape measure protein and is found in phages Genki and Gryphonian, but not among the remaining four phages in the subcluster, all of whom have ~99% ANI with Genki and Gryphonian (Supplemental Figure S1). 

This evidence suggests the existence of one or more point mutations in the protospacer sequence. We searched the genomes of Fern cluster phages that lacked protospacers 3, 7, 15, and 16 for mutations at that location by aligning the protospacer sequences with the phage genomes. Of the 11 Fern cluster phages not containing the exact sequence of protospacer 3, ten of them were found to have a single point mutation in the protospacer region, while the same region in phage BN12 differed by five nucleotides. All of the 26 Fern cluster phages that are missing protospacer 16 were found to have one or two point mutations in the protospacer region. This was not the case for protospacers 7 and 15; Fern cluster phages that are missing these protospacers have more than 10 nucleotides sequence differences in the protospacer region.

The full list of the 18 unique protospacers, their length, the *P. larvae* strains, phages, and phage genes they are found in, is shown in Table 4. Protospacer length ranges from 33 bp to 38 bp. Sixteen protospacers are located in coding regions; two are located in intergenic regions (*P. larvae* phage genomes are 90–95% coding, [25]). Of the 16 protospacers located in coding regions, eight are located in a gene with putative function, and eight in a hypothetical gene (about half of *P. larvae* genes have putative function, [25]). Protospacer 3 is located in the large terminase gene (near the genome start), and protospacer 16 in the tail tape measure gene; both genes are conserved in *P. larvae* phages [25]. Both of these protospacers are found in the SAG 10367 strain (ERIC II). Nevertheless, no pattern is discernible regarding which part of the phage genome the protospacers are recruited from; protospacers are recruited in the front, middle, and rear of the phage genome, and in genes of widely differing functions, as well as hypothetical proteins and intergenic regions. For example, protospacer 11 is located between bases 72–105 in a hypothetical gene in phage API 480, while protospacer 10 is located in a hypothetical gene at the tail end (bases 51,647–51,683) of the genome of phage Tripp. 

**Figure 3:**
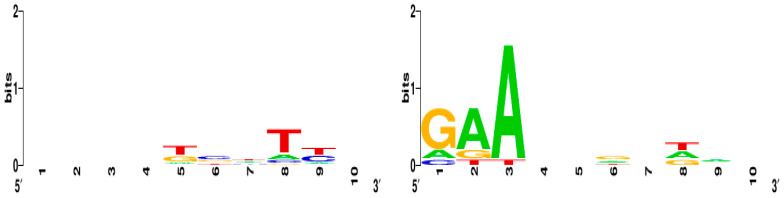
Sequence logo of the 10 bases upstream and the 10 bases downstream of the protospacers. The -GA(A)- sequence in the 10 bases downstream is likely the PAM sequence and is downstream of 17 of the 18 protospacers.

We identified a -GA(A)- sequence in the 10 downstream bases in 17 out of 18 protospacers that is a likely PAM sequence. No PAM sequence was identified in the 10 upstream bases. Logos of the putative PAM sequences are shown in Figure 3. The 10 bases upstream and downstream of the protospacers are included as Supplemental Table S2.

## 4. Discussion

This study establishes the existence of CRISPR spacer and protospacer sequences in the genomes of sequenced strains of *P. larvae* and *P. larvae* phages. Searching the genomes of nine *P. larvae* strains, we identified 384 unique spacer sequences. The number of spacers per strain ranges from 7 to 169, which is similar to what has been observed in systems such as *Clostridium difficile* (43–153 spacers per strain) [49], and *Microcystis aeruginosa* (47–174 spacers per strain) [50]. Of importance is that the epidemiologically important ERIC I strains ATCC 9545 and DSM 7030, and the ERIC II strain DSM 25340 contain relatively few spacers (fewer than 20); presumably, AFB outbreaks caused by these or related strains would be the most treatable with phages. In general, the ERIC I and ERIC II strains contain an order of magnitude fewer spacers than the ERIC III–V strains, the sole exception to this being the ERIC II strain SAG 10367, which contains the highest number of spacers (169). It is known that CRISPR-Cas and horizontal gene transfer (HGT) oppose one another; an increase in the frequency of one results in a decrease in the frequency of the other. While the ERIC I strains are responsible for the majority of AFB outbreaks globally, the ERIC III–V strains are vanishingly rare in the field and exist mostly in archived cultures; they would thus presumably not experience as much HGT as the ERIC/II strains. Thus, one possible explanation for the low frequency of spacers in the ERIC I strains is that these strains downregulate CRISPR-Cas so as to facilitate acquisition of beneficial genes through HGT, whereas the ERIC III and ERIC IV strains would have less need for HGT. On the other hand, the high number of spacers in the SAG 10367 strain implies that this strain frequently comes under attack by phages, resulting in a large CRISPR array.

Approximately two-thirds (66%) of spacers are unique to a *P. larvae* strain, suggesting distinct acquisition events. The main exception to this are the four genomically similar ERIC III and IV strains, which largely share the same spacers due to common descent. By comparison, the percentage of spacers that are unique to a strain ranges from as low as 9% in *Escherichia coli* [51], to 75% for *Vibrio cholerae* [52], and 98% for the genus *Thermus* [53]. This suggests that the genomic diversity of *P. larvae* still remains to be fully sampled, although not to the extent of non-culturable genera such as *Thermus*.

Of the 384 unique spacers, only 18 (~5%) were found in the 49 sequenced *P. larvae* phage genomes as protospacers. This low coverage implies the existence of a large number of novel undiscovered *P. larvae* phages, and that the bulk of the genetic landscape of *P. larvae* phages remains to be discovered. By comparison, spacer coverage in *C. difficile* ranges from 17% to 38%, with 162 unique protospacer sequences in 31 phages and prophage genomes [49], while a study of the *Vibrio cholerae* system found 34% protospacer coverage [52]. On the other hand, the protospacer coverage of *P. larvae* phages is similar to what has been reported for phages that infect less intensively studied hosts, such as *Microcystis aeruginosa* (~4% coverage) and the genus *Thermus* (6% coverage) [50,53]. It has similarly been proposed that the bulk of the genetic landscape of *Thermus* phages is undiscovered for the same reason [53].

The distribution of the 18 protospacer sequences is uneven among *P. larvae* strains. Strain SAG 10367 (ERIC II) contains eight unique protospacers from every phage cluster or singleton, while strain ATCC 9545 (ERIC I) does not contain any protospacers and strains DSM 7030 (ERIC I) and DSM 25430 (ERIC II) contain only one protospacer. The low number of protospacers in three of the four ERIC I/II strains is encouraging for the use of *P. larvae* phages to treat AFB. On the other hand, we should expect that AFB outbreaks caused by strain SAG 10367, or strains related to it, to be the most difficult to treat with phages. As with the spacers, most protospacers are unique to a *P. larvae* strain, the sole exception being the protospacers shared between the ERIC III and ERIC IV strains due to genetic relatedness. The fact that the majority of protospacers are unique to a particular strain could explain why phages whose spacers are found in *P. larvae* are still able to lyse; presumably, the strain of *P. larvae* they are able to lyse is a different strain from the one containing their protospacer.

The protospacers are generally unique to individual phages as well, or else small groups of very closely related phages; only four out of 18 protospacers do not fit this pattern, and only one protospacer is found in two different phage clusters. Approximately a third of the 49 sequenced *P. larvae* phages do not contain any protospacers sequences at all; this is particularly encouraging for the use of phages to treat AFB, and such phages should be preferred in phage cocktails used to treat infected beehives. No phages contain more than four protospacers, which is a pretty low number. No pattern is discernible regarding where the protospacers are recruited from in the phage genomes; protospacers are found to originate from conserved genes, non-conserved genes, hypothetical proteins, as well as intergenic regions. In contrast, all *C. difficile* phages were found to contain anywhere from one to 16 protospacers, every *C. difficile* strain had at least one spacer from a phage, and the spacers were noticeably recruited from conserved genes [49].

All sequenced *P. larvae* phages are strongly lytic in vitro [22,25], including those phages that contain protospacer sequences identified in this study. For example, phages Fern and Willow are among the most strongly lytic phages [13], but at least one *P. larvae* strain (SAG 10367 of the ERIC II genotype) contains a protospacer sequence from their large terminase protein. Similarly, three protospacer sequences were found in the genome of phage Harrison, but this phage is also one of the most strongly lytic *P. larvae* phages [13]. Though the present data is sparse, there does not appear to be a negative correlation between presence of protospacers in the phage genome and lytic ability. This also raises the question of whether *P. larvae* phages evade their host’s CRISPR defenses by means of anti-CRISPR genes, particularly considering that currently half of *P. larvae* phage proteins do not have putative function [25]. A preliminary search for anti-CRISPR genes using AcrFinder did not yield results [54], but more work remains to be done in this area.

An additional mechanism by which *P. larvae* phages may evade CRISPR defense systems is by point mutations in the protospacer or PAM sequence [55]. While 17 of the 18 protospacers appear to contain the putative -GA(A)- PAM sequence, two protospacers, one located in the conserved large terminase gene (protospacer 3), and one located in the conserved tail tape measure gene (protospacer 16), were found to have possible point mutations at one or two locations in their protospacer sequence. This could be direct evidence of the evolutionary arms race between *P. larvae* and their phages. However, for phages to be able to escape CRISPR through mutation, the mutation has to be in the seven-base “seed” sequence of the protospacer [56]; it is not yet known if the putative point mutations we identified in the phage protospacers are indeed in the seed region.

The existence of CRISPR protospacers in a phage genome is an important consideration when selecting phages for therapy, whether to treat AFB in honeybees or infections in other organisms. A recent study by Philipson et al. describes a thorough workflow for selecting phages for therapeutic applications [57]. To this workflow we would add the following: use CRISPRFinder to identify spacer sequences in the host, then search the candidate therapeutic phage for the spacer sequences; preference for therapeutic applications should be given to those phages without protospacers in the host genome, or if that is not possible, phages with protospacers from the same host. For example, under this scheme phage Halcyone (no protospacers) would be a better choice for treating AFB than phage Saudage (three protospacers from the same strain), which would in turn be a better choice than phages Harrison and Paisley (three protospacers, each from a different strain of *P. larvae*).

As interest in *P. larvae* phages continues to grow, the number of sequenced *P. larvae* strains and phages will grow as well. It will be interesting to obtain a more complete picture of the genomic and CRISPR landscape of these phages, especially with regards to the existence of anti-CRISPR genes in their genomes or other means they use to evade host defenses. Additional future directions include testing experimentally the ability of *P. larvae* phages to lyse *P. larvae* strains that contain protospacers from the phages, and more detailed and comprehensive comparisons with the distribution of spacer and protospacer sequences in other phage–host systems.

## 5. Conclusions

We present the first analysis of CRISPR spacer sequences identified in nine sequenced *P. larvae* strains and 49 sequenced phages. Three of the four commercially important *P. larvae* strains contain few spacers and protospacers, which is a positive finding for phage therapy of AFB. Moreover, approximately a third of phages do not contain any protospacers, an additional third contains only one protospacer, and the most protospacers in a phage genome is four. Protospacers are thus relatively scarce in the *P. larvae* system, with only 5% of spacers doubling as protospacers. This is an encouraging finding for phage therapy, and also implies that much of the genomic landscape of *P. larvae* phages remains undiscovered. Some phages appear to have point mutations in their protospacer sequences, possibly so as to evade the hosts’ CRISPR defenses. The results of this study serve as a marker for future studies on the CRISPR-Cas system in *P. larvae* as well as in other host–phage systems.

## Figures and Tables

**Table 1:** CRISPR array and spacer data for the *P. larvae* strains with a completely sequenced genome used in this study.

*P. larvae* Strain	No. of CRISPR Arrays	No. of Spacers	No. of Unique Spacers	*P. larvae* Genotype	GenBank Accession Number
**ATCC 9545**	4	17	17	ERIC I	CP019687
**DSM 7030**	4	17	17	ERIC I	CP019651
**DSM 25430**	1	8	5	ERIC II	CP003355
**SAG 10367**	7	169	159	ERIC II	CP020557
**LMG 16252**	6	95	93	ERIC III	CP019655
**ATCC 13537**	7	97	95	ERIC IV	CP019794
**CCM 38**	7	97	95	ERIC IV	CP020327
**LMG 16247**	7	98	96	ERIC IV	CP019659
**DSM 106052**	6	116	111	ERIC V	CP019717

**Table 2:** Frequency of occurrence of spacers in *P. larvae* strains.

	1 Strain	2 Strains	3 Strains	4 Strains	5 Strains	6 Strains	8 Strains
**No. of spacers found in**	254	30	12	83	2	1	2
**% of spacers found in**	66.1%	7.8%	3.1%	21.6%	0.5%	0.25%	0.5%

**Table 3:** Number of prophages in *P. larvae* strains and number of spacers located in prophage regions.

*P. larvae* Strain	No. of Prophages			No. of Spacers Located in Prophages		
	Intact	Questionable	Incomplete	Intact	Questionable	Incomplete
**ATCC 9545**	5	2	10	0	0	0
**DSM 7030**	5	1	12	0	0	0
**DSM 25430**	2	1	9	0	0	0
**SAG 10367**	5	7	12	0	0	1
**LMG 16252**	8	3	7	0	1	0
**ATCC 13537**	5	3	6	0	0	1
**CCM 38**	6	3	11	0	1	1
**LMG 16247**	4	5	8	0	2	0
**DSM 106052**	5	5	14	0	0	0

**Table 4:** Complete list of the 18 unique protospacer sequences found in *P. larvae* strains and phages.

	Protospacer Sequence	Protospacer Length (bp)	Strains Containing Protospacer	Phages Containing Protospacer	Gene Containing Protospacer
**1**	AACAATTACAAATATGCAACTGAAGCAGATGTAAAT	36	DSM 7030	Harrison Paisley	ERF superfamily single-stranded DNA binding
**2**	AACGATTTTACAACGATTATAACACGTAAATACAAG	36	SAG 10367	Tripp	Intergenic
**3**	AGAAAAACTGGACGGGTTAAACACACATTTGGCG	34	SAG 10367	Arcticfreeze, Bloom, DevRi, Genki, Gryphonian, HB10c2, Honeybear, Jacopo, Kiel007, Likha, Lucielle, Pagassa, PBL1c, Redbud Saudage, Tadhana Toothless, Xenia, Yerffej	Large Terminase
**4**	AGCACATAAGTAAAGGAATACCCCCGGCTCTGGACATT	38	DSM 106052	Ash C7Cdelta Ley	Hypothetical
**5**	ATCCGGTGCATCAGAGATTGGCTCAACTGTATTTCAA	37	ATCC 13537 CCM 38 LMG 16247 LMG 16252	LincolnB Wanderer	Hypothetical
**6**	CAGAAGTACCCCTTGGGACATATGATGTGAAGATT	35	ATCC 13537 CCM 38 LMG 16247 LMG 16252	LincolnB Wanderer	Hypothetical
**7**	CGAACATATCCGGAGTCAACTATATCAGACTCACTCA	37	SAG 10367	Fern, Kawika, Lily, Lucielle, Saudage, Willow	Replicative DNA helicase
**8**	GAATTTGTAAAAGTTCTACAAGATGAAGATATTAC	35	ATCC 13537 CCM 38 LMG 16247 LMG 16252	Lily	Hypothetical
**9**	GAGCAAGCTGCAACAGAACCGAAATGGACCACT	33	DSM 106052	LincolnB Wanderer	Hypothetical
**10**	GGAAACTGGCGAGCGCATCGTATGGGGGACTGCATCG	37	SAG 10367	Tripp	Hypothetical
**11**	GGAAATGATGGAGAGATACATAGAGCATTTGCCA	34	SAG 10367	API480	Hypothetical
**12**	GGAAGCTGACCGAAAGAGACTAATCGCCGTACAAGA	36	DSM 106052	Ash, C7Cdelta, Ley, Tripp	DNA polymerase
**13**	GTGCTTGACCACATGGGGGCATTCATGGAAACA	32	DSM 106052	Lily	Baseplate
**14**	GTTAGACGAGCGTGTGAGGAGGCTGCAACAGGCA	34	SAG 10367	Harrison, Paisley	DNA replication
**15**	TCCCTACCAAAAGGAGGGTAGGATTAGTGGAAGTT	35	CCM 38 LMG 16247 LMG 16252	Kawika	Intergenic
**16**	TCTAGAAGCCATTGTCAAAAAAATCACGGAAGTGTT	36	SAG 10367	Lucielle, Genki, Gryphonian, PBL1c	Tail Tape Measure
17	TGCGGAGGGCAATCCCAACAGACTGACGAAAGAA	34	SAG 10367	Dragolir, LincolnB Wanderer	Small Terminase
18	TTACAGGGGCAGGGAGGTACAGAAGATAGGAGGTAC	36	DSM 25430	Harrison, Paisley	Hypothetical

## Data Availability

All *P. larvae* and *P. larvae* phage genome sequences are available on NCBI GenBank; the phages’ GenBank accession numbers are listed in Ref. 25. The full list of spacers identified in the study is included as Supplementary Table S1.

## Supplementary Materials

The following are available online at https://www.mdpi.com/1999-4915/13/3/459/s1, Table S1: Full list of spacer sequences identified in the *Paenibacillus larvae* genome; Table S2: 10 bp sequences upstream and downstream of the spacers; Figure S1: ANI matrix of the 49 sequenced *P. larvae* phages.
