# Supplementary figures and images for: Characterization of CRISPR Spacer and Protospacer Sequences in Paenibacillus larvae and Its Bacteriophages
Source: Viruses. 2021 Mar 11;13(3):459. doi: 10.3390/v13030459 (PMC7998209; doi:10.3390/v13030459)

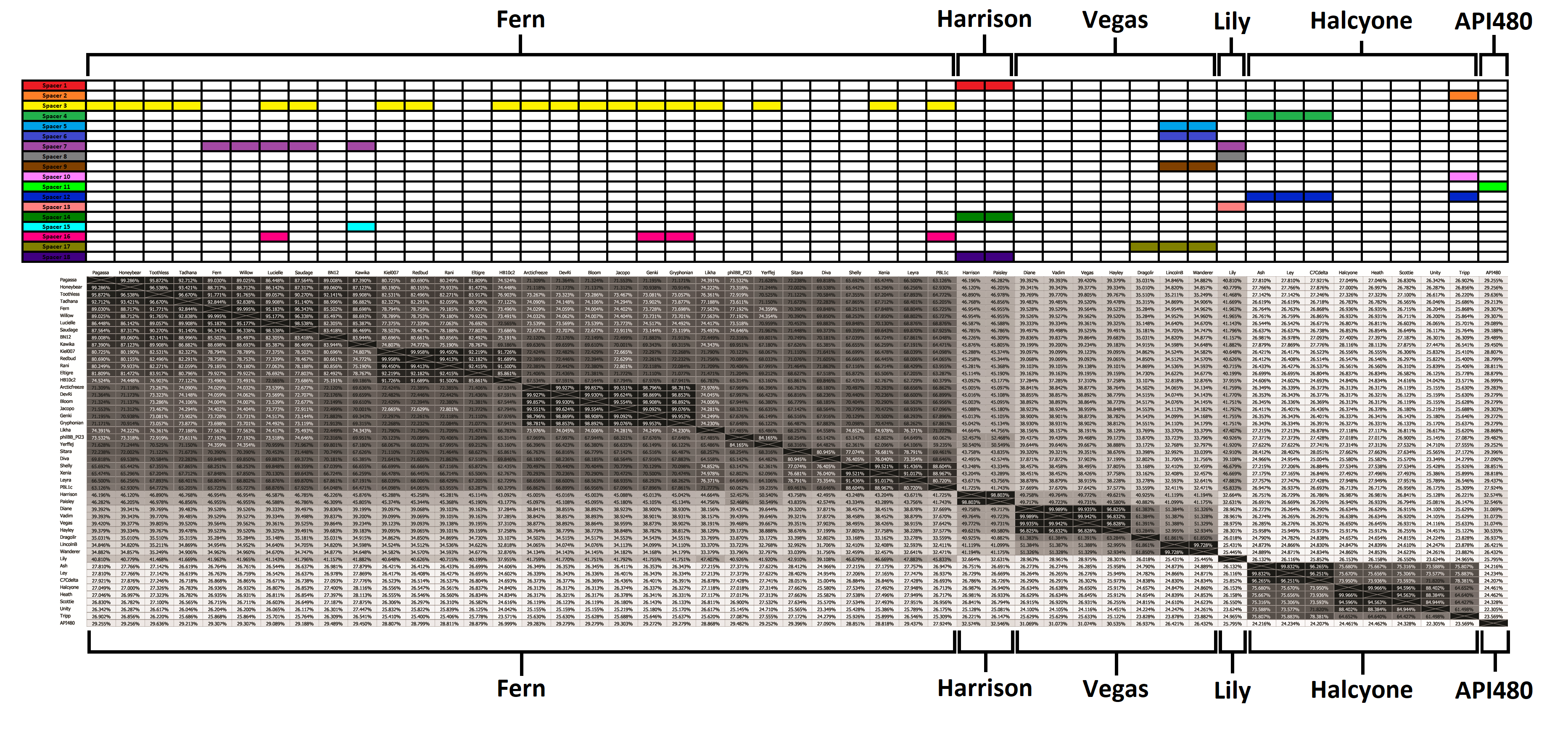

Supplement: Supplementary file 1 [file viruses-13-00459-s001.zip › viruses-1099430-supplementary/Supplementary Figure 1.tif]
